# Supplementary figures and images for: Comparative genome analysis of Salmonella enterica serovar Gallinarum biovars Pullorum and Gallinarum decodes strain specific genes
Source: PLoS One. 2021 Aug 19;16(8):e0255612. doi: 10.1371/journal.pone.0255612 (PMC8375982; doi:10.1371/journal.pone.0255612)

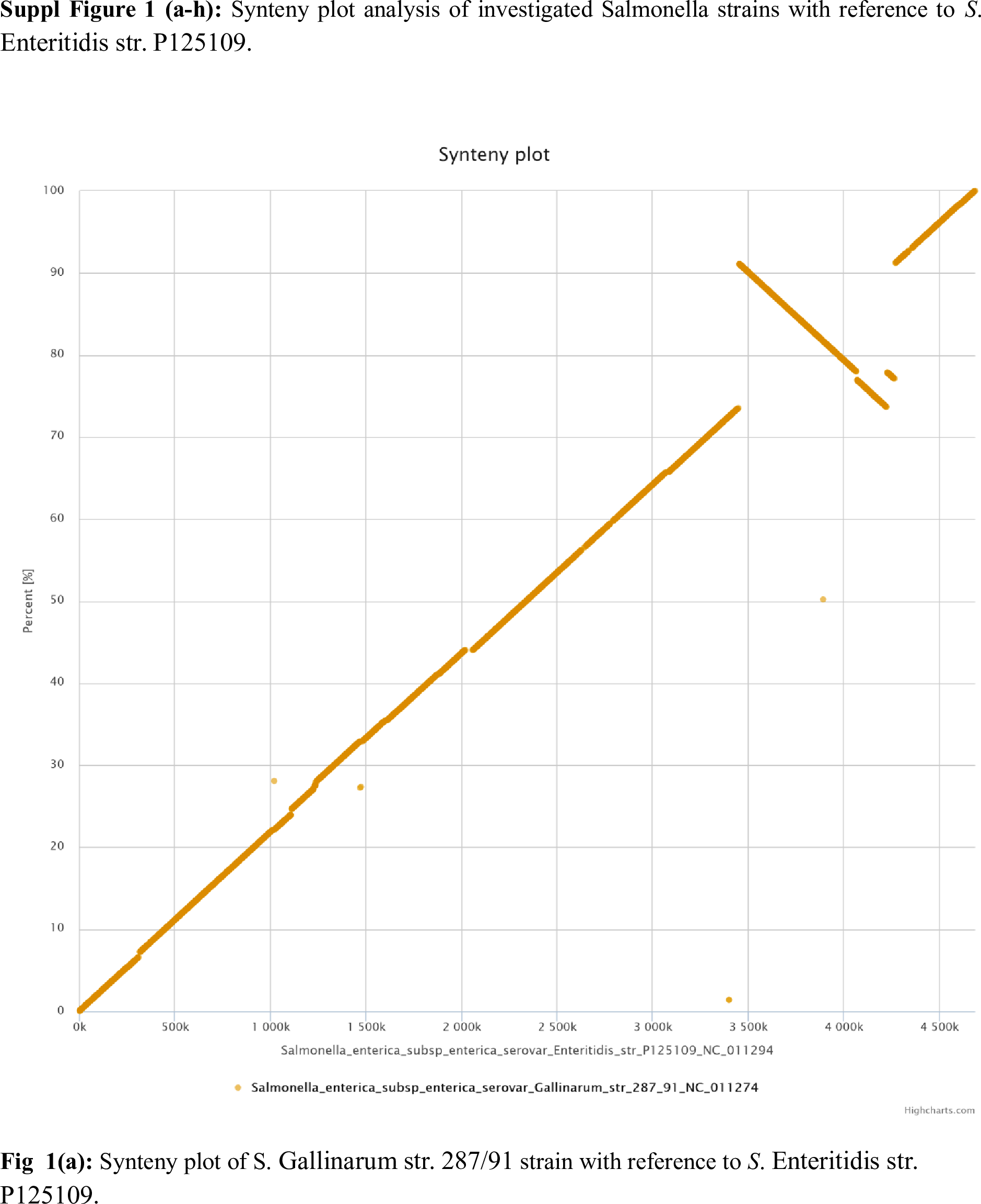

Supplement: S1 Fig — (a-h) Synteny plot analysis of investigated Salmonella strains with reference to S. Enteritidis str. P125109. Depicts synteny plot analysis of investigated Salmonella strains. (TIFF) [file pone.0255612.s001.tiff]

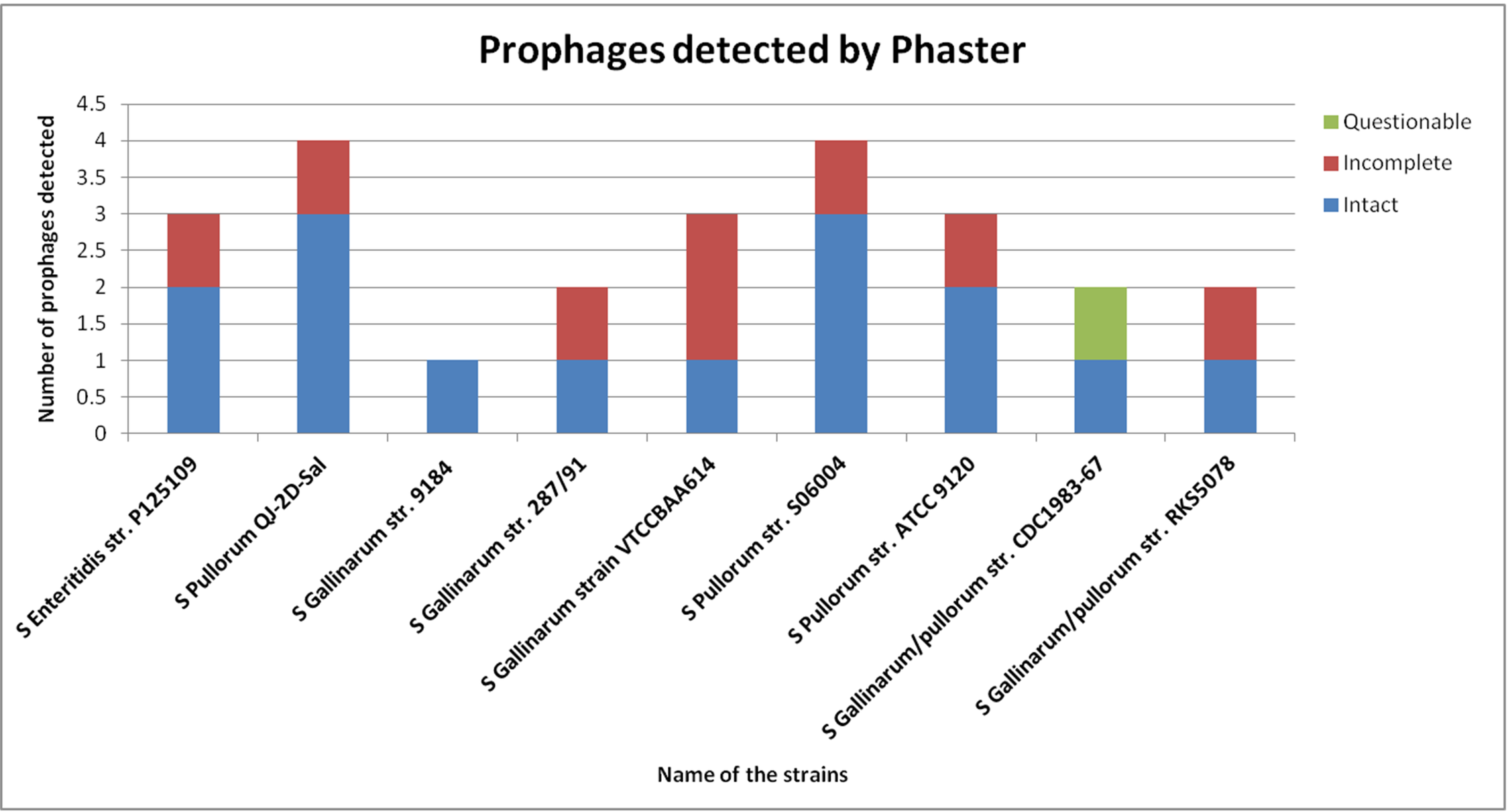

Supplement: S2 Fig — Details of prophages detected. (TIF) [file pone.0255612.s002.tif]

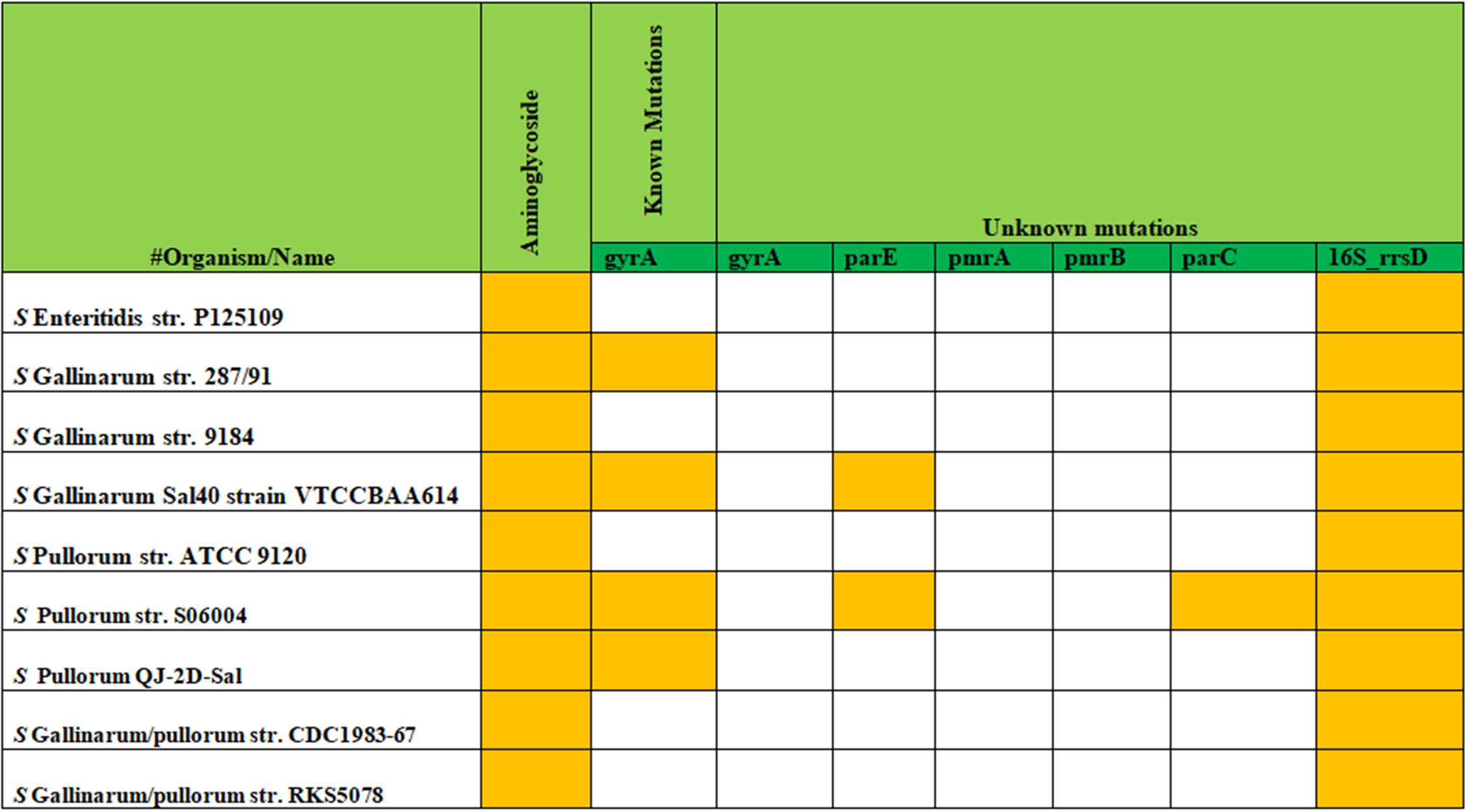

Supplement: S3 Fig — Detects acquired antimicrobial resistance genes. (TIF) [file pone.0255612.s003.tif]
