# Supplementary material for: Comparative genome analysis of Salmonella enterica serovar Gallinarum biovars Pullorum and Gallinarum decodes strain specific genes
Source: PLoS One. 2021 Aug 19;16(8):e0255612. doi: 10.1371/journal.pone.0255612 (PMC8375982; doi:10.1371/journal.pone.0255612)
Supplement: S1 Table — Pan-genome and core genome development projections. (DOCX) [file pone.0255612.s004.docx]

**S1 Table.** Pan-genome and core genome development projections for investigated nine *Salmonella* strains

| **Pan-genome development extrapolations** | | **Core genome development extrapolations** | |
| --- | --- | --- | --- |
| **Contigs** | **Mean number of genes** | **Contigs** | **Mean number of genes** |
| **1** | 4186.444 | **1** | \| 4183.889 \| \| --- \| |
| **2** | 4458.750 | **2** | 3914.139 |
| **3** | 4597.726 | **3** | 3778.988 |
| **4** | 4707.119 | **4** | 3673.198 |
| **5** | 4800.254 | **5** | 3580.563 |
| **6** | 4882.595 | **6** | 3495.643 |
| **7** | 4957.333 | **7** | 3416.194 |
| **8** | 5026.444 | **8** | 3341.222 |
| **9** | 5091.000 | **9** | 3270.000 |
| **Heap’s law extrapolation** | | **Heap’s law extrapolation** | |
| Growth exponent y:  0.089 (alpha 0.911) | | Converges to:  2684.387 | |
| Confidence interval (95%) for y:  0.084 to 0.093 | | Confidence interval (95%):  from 2507.663 to 2861.111 | |
| Fitted model:  4174.444 * x^0.089 | | Fitted model:  1506.312 * exp(-x/9.612) + 2684.387 | |
| Lower confidence interval formula:  4142.266 * x^0.084 | | Lower confidence interval formula:  1358.388 * exp(-x/7.656) + 2507.663 | |
| Upper confidence interval formula:  4206.622 * x^0.093 | | Upper confidence interval formula:  1654.236 * exp(-x/11.569) + 2861.111 | |
